# Predictors of diffuse alveolar damage in patients with acute respiratory distress syndrome: a retrospective analysis of clinical autopsies

**DOI:** 10.1186/s13054-017-1852-5

**Published:** 2017-10-20

**Authors:** Arnaud W. Thille, Oscar Peñuelas, José A. Lorente, Pilar Fernández-Segoviano, José-Maria Rodriguez, José-Antonio Aramburu, Julian Panizo, Andres Esteban, Fernando Frutos-Vivar

**Affiliations:** 1 CHU de Poitiers, Réanimation Médicale, Poitiers, France; 2 INSERM CIC 1402 ALIVE Group, Université de Poitiers, 2 rue la Milétrie, 86021 Poitiers Cedex, France; 3 Departamento de Cuidados Intensivos, Hospital Universitario de Getafe, Madrid, Spain; 4 CIBER de Enfermedades Respiratorias, Universidad Europea de Madrid, Madrid, Spain; 5 Departamento de Anatomía Patológica, Hospital Universitario de Getafe, CIBER de Enfermedades Respiratorias, Madrid, Spain; 6 Departamento de Radiodiagnóstico, Hospital Universitario de Getafe, Madrid, Spain

**Keywords:** Acute respiratory distress syndrome, Diffuse alveolar damage, Mechanical ventilation, Intensive care unit

## Abstract

**Background:**

Although diffuse alveolar damage (DAD) is considered the typical histological pattern of acute respiratory distress syndrome (ARDS), only half of patients exhibit this morphological hallmark. Patients with DAD may have higher mortality than those without DAD. Therefore, we aimed to identify the factors associated with DAD in patients with ARDS.

**Methods:**

We analyzed autopsy samples of 356 patients who had ARDS at the time of death. DAD was assessed by two pathologists, and ARDS criteria were evaluated by two intensivists. Criteria for severe ARDS included the degree of hypoxemia and the ancillary variables of the current Berlin definition assessed within 48 h before death: radiographic severity, high positive end-expiratory pressure (PEEP) level, and physiological variables (i.e., altered respiratory system compliance and large anatomic dead space).

**Results:**

After multivariable analysis, high PEEP levels, physiological variables, and opacities involving only three quadrants on chest radiographs were not associated with DAD. The four markers independently associated with DAD were (1) duration of evolution (OR 3.29 [1.95–5.55] for patients with ARDS ≥ 3 days, *p* < 0.001), (2) degree of hypoxemia (OR 3.92 [1.48–10.3] for moderate ARDS and 6.18 [2.34–16.3] for severe ARDS, *p* < 0.01 for both), (3) increased dynamic driving pressure (OR 1.06 [1.04–1.09], *p* = 0.007), and (4) radiographic severity (OR 2.91 [1.47–5.75] for patients with diffuse opacities involving the four quadrants, *p* = 0.002). DAD was found in two-thirds of patients with a ratio of partial pressure of arterial oxygen to fraction of inspired oxygen ≤ 100 mmHg and opacities involving the four quadrants.

**Conclusions:**

In addition to severe hypoxemia, diffuse opacities involving the four quadrants were a strong marker of DAD.

## Background

Acute respiratory distress syndrome (ARDS) was described for the first time in 1967 by Ashbaugh and colleagues [1]. They reported 12 patients without a history of underlying cardiac or pulmonary disease who rapidly developed acute hypoxemia, stiff lungs, and diffuse opacities visualized by chest radiography. An autopsy was performed in the seven deceased patients, and a striking finding was the presence of hyaline membranes in all but one patient [1]. Diffuse alveolar damage (DAD), including the presence of hyaline membranes, interstitial edema, cell necrosis and proliferation, or fibrosis, is considered the typical histological pattern of ARDS [2, 3]. However, this histological hallmark of DAD is not observed in all patients fulfilling the criteria for ARDS. In the literature, the proportion of DAD among patients with ARDS ranged from 45% to 66% in autopsy studies [4–7] and from 14% to 60% in open lung biopsy studies [8–14]. Patients without DAD exhibited other histological patterns, such as diffuse interstitial pneumonia, pulmonary infarction or hemorrhage, lymphangitis, cancer infiltration, bacterial pneumonia without DAD, or no lung histological abnormalities at all.

In a recent meta-analysis including all patients with ARDS who had open lung biopsy, only 51% of them had DAD at histological examination [15]. This meta-analysis showed that patients with ARDS and DAD had higher mortality than those without DAD. In a previous study analyzing autopsy findings from patients with ARDS according to the Berlin definition, DAD proportion was correlated to degree of hypoxemia and was 12% in mild ARDS, 40% in moderate ARDS, and 58% in severe ARDS [7]. Whether other markers could be associated with the presence of DAD in patients with ARDS has been poorly assessed. Their identification could contribute to better management of ARDS. We consequently aimed to identify markers associated with DAD from clinical autopsies in patients fulfilling the criteria for ARDS at the time of death.

## Methods

This is a secondary analysis of a large database of clinical autopsies [7].

### Inclusion of patients

We analyzed the 356 patients with ARDS who died and had a clinical autopsy between 1 January 1991 and 31 December 2010 in the 18-bed intensive care unit (ICU) at the University Hospital of Getafe in Spain. We systematically requested informed consent from patients’ relatives for both clinical autopsy and potential use of tissue samples for subsequent data analysis in research or teaching purposes, and the study was approved by the institutional review board.

All medical files of patients having had an autopsy were reviewed by two intensivists blinded to the autopsy findings to determine if patients fulfilled the clinical criteria for ARDS at the time of death. The diagnosis of ARDS was established by consensus of the two intensivists and resolved by a third intensivist in case of discrepancies. According to the Berlin definition [16], patients were considered to have the diagnosis of ARDS if they had (1) acute respiratory failure not fully explained by cardiac failure or fluid overload; (2) bilateral opacities not fully explained by effusions, lobar/lung collapse, or nodules on the chest radiograph or the CT scan; and/or (3) onset within 1 week after a known clinical insult or new/worsening respiratory symptoms.

### Data collection and definition of severity

We collected demographic variables (age, sex, severity score at ICU admission), reasons for ICU admission and mechanical ventilation, duration of mechanical ventilation, and date of the onset of risk factor for ARDS. Patients were considered to have a pulmonary risk factor for ARDS if they had a diagnosis of pneumonia, aspiration, inhalation, or lung contusion, as well as an extrapulmonary risk factor for ARDS if they had a diagnosis of sepsis, shock, multiple trauma or transfusion, or pancreatitis. Severity was defined according to degree of hypoxemia, and ARDS was considered severe if the ratio of partial pressure of arterial oxygen to fraction of inspired oxygen (PaO_2_/FiO_2_) was ≤ 100 mmHg, moderate if PaO_2_/FiO_2_ was between 101 and 200 mmHg, and mild if PaO_2_/FiO_2_ was between 201 and 300 mmHg, in all cases using a PEEP level ≥ 5 cmH_2_O.

We also distinguished the more severe patients according to the four ancillary variables proposed by the Berlin definition for severe ARDS: (1) radiographic severity with more extensive opacities involving three or four quadrants compared with patients fulfilling the minimal criterion of “bilateral opacities” (two quadrants), altered respiratory system compliance (C_RS_ ≤ 40 ml/cmH_2_O), large anatomical dead space as indicated by high corrected expired volume per minute (≥10 L/minute), and positive end-expiratory pressure (PEEP) ≥ 10 cmH_2_O. Because plateau pressure was not measured over the whole period of the study, we calculated dynamic driving pressure (expressed as centimeters of water) as the difference between peak inspiratory pressure minus PEEP, and dynamic compliance of the respiratory system, and not static C_RS_, as tidal volume divided by peak inspiratory pressure minus PEEP and expressed in milliliters per centimeter of water. Corrected expired volume per minute was calculated as the measured minute ventilation multiplied by the partial pressure of arterial carbon dioxide (PaCO_2_) divided by 40 mmHg.

In all patients with ARDS, we collected ventilatory settings, arterial blood gas results, and chest radiography findings within 48 h before death. Radiographic severity was assessed by two intensivists, and extent of opacities was classified according to the number of quadrants involved (two, three, or all four). In case of disagreement, discrepancies were resolved by a consensus of three physicians, including a third intensivist or a radiologist blinded to the autopsy findings. Patients with four quadrants involved were those with bilateral and diffuse infiltrates without pleural effusion or lung collapse.

### Pathologic criteria for DAD

All our autopsy examinations were performed within 12 h of death using the same methodology for over 20 years, as already detailed in our previous studies in this field of investigation [4, 7, 17–19]. After removal from the thorax, the lungs were inflated at a pressure of 35 cmH_2_O and were fixed in blocks with 10% formalin. We took samples for microscopic analysis from each pulmonary lobe and additional samples from areas with macroscopic injuries. All samples were stored from the time of autopsy and were subsequently independently analyzed by two pathologists who had no clinical information. A third pathologist, also blinded, resolved any discrepancies. Criteria for DAD were hyaline membranes plus at least one of the following: intraalveolar edema, cell necrosis of type I alveolar cells, proliferation of type II alveolar cells (cuboidal cells), interstitial proliferation of fibroblasts and myofibroblasts, or organizing interstitial fibrosis [2, 3, 17].

### Statistical analysis

Patients with ARDS and DAD and those with ARDS without DAD were compared using the chi-square test or Fisher’s exact test for categorical variables and Student’s *t* test or the Mann-Whitney *U* test for continuous variables when appropriate. Continuous variables were expressed as mean ± SD or as median and IQR (25th–75th percentiles). Qualitative variables were expressed as frequency and percent.

To identify independent factors associated with DAD, we performed a multivariate logistic regression analysis using a backward selection procedure including in the maximal model all nonredundant variables associated with DAD with a *p* value < 0.15.

A two-tailed *p* value < 0.05 was considered statistically significant. All analyses were performed using SAS version 9.2 software (SAS Institute, Cary, NC, USA).

## Results

Among the 356 patients analyzed who fulfilled the criteria for ARDS at the time of death, only 45% of them (159 patients) had DAD at autopsy examination, whereas 8% (27 patients) had normal lungs. Using univariate analysis (Table 1), patients with DAD were more likely than those without DAD to have pneumonia as the lung insult triggering ARDS and also to be female, although they received lower tidal volumes than males (580 ± 135 vs. 644 ± 143 ml, *p* < 0.001). Patients with DAD were more hypoxemic and more hypercapnic. They also had more increased dynamic driving pressure, and all ancillary variables proposed for severe ARDS in the Berlin definition were associated with DAD (Table 1). Patients with DAD had more altered compliance of the respiratory system, larger anatomical dead space as indicated by higher expired volume per minute corrected by PaCO_2_, higher PEEP levels, and more diffuse opacities on chest radiographs. Discrepancies of chest radiographic analysis concerning the number of quadrants involved were found in 19% of the cases (*n* = 69) and were resolved by a consensus of three physicians. At autopsy examination, the lungs from patients with DAD were heavier than those without DAD.

**Table 1:** Comparison of patients with acute respiratory distress syndrome with or without diffuse alveolar damage patterns at histological examination

	ARDS without DAD (*n* = 197)	ARDS with DAD (*n* = 159)	*p* Value
Characteristics of the patients			
Age, years	66.4 ± 14.0	64.3 ± 14.7	0.17
Female sex, *n* (%)	58 (29.4%)	68 (42.8%)	0.01
SAPS II, points	55.5 ± 19.6	50.9 ± 16.1	0.02
Duration of MV before death, days	12.7 ± 21.7	13.1 ± 11.0	0.82
Duration of ARDS evolution ≥ 72 h, *n* (%)	101 (51%)	128 (81%)	<0.01
Risk factor triggering ARDS			
Pneumonia, *n* (%)	62 (31.5%)	66 (41.5%)	0.04
Other pulmonary risk factors of ARDS, *n* (%)	12 (6.1%)	7 (4.4%)	0.48
Shock, *n* (%)	118 (59.9%)	83 (52.2%)	0.14
Extrapulmonary sepsis, *n* (%)	73 (37.1%)	56 (35.2%)	0.72
Pancreatitis, *n* (%)	12 (6.1%)	7 (4.4%)	0.48
Blood gases			
PaO_2_, mmHg	93 ± 37	79 ± 27	<0.01
PaO_2_/FiO_2_, mmHg	140 ± 67	104 ± 53	<0.01
PaCO_2_, mmHg	44.5 ± 12.8	49.1 ± 15.0	<0.01
Severity of hypoxemia			<0.01
200 < PaO_2_/FiO_2_ ≤ 300 mmHg, *n* (%)	43 (22%)	6 (4%)	
100 < PaO_2_/FiO_2_ ≤ 200 mmHg, *n* (%)	85 (43%)	56 (35%)	
PaO_2_/FiO_2_ ≤ 100 mmHg, *n* (%)	69 (35%)	97 (61%)	
Ventilatory settings			
Tidal volume, ml	620 ± 133	623 ± 157	0.87
Respiratory rate, cycles/minute	20 ± 6	22 ± 7	<0.01
Minute ventilation, L/minute	11.9 ± 3.2	13.1 ± 3.4	<0.01
FiO_2_	0.75 ± 0.25	0.85 ± 0.22	<0.01
PEEP, cmH_2_O	7.9 ± 4.5	9.6 ± 4.1	<0.01
PEEP ≥ 10 cmH_2_O, *n* (%)	91 (46%)	106 (67%)	<0.01
Physiological parameters			
Peak pressure, cmH_2_O	37 ± 10	44 ± 10	<0.01
Dynamic driving pressure (peak − PEEP), cmH_2_O	28 ± 11	34 ± 9	<0.01
Dynamic compliance, ml/cmH_2_O	24 ± 11	20 ± 7	<0.01
Dynamic compliance < 40 ml/cmH_2_O, *n* (%)	174 (88%)	155 (97%)	<0.01
VE corrected PaCO_2_, L/minute	13 ± 5	16 ± 7	<0.01
VE corrected PaCO_2_ ≥ 10 L/minute, *n* (%)	135 (69%)	133 (84%)	<0.01
Extent of infiltrates on chest radiograph			<0.01
Two quadrants, *n* (%)	64 (32%)	17 (11%)	
Three quadrants, *n* (%)	63 (32%)	41 (26%)	
Four quadrants, *n* (%)	70 (36%)	101 (63%)	
Lung weight at autopsy examination, g	1567 ± 709	1848 ± 748	<0.01

*Abbreviations*: *ARDS* Acute respiratory distress syndrome, *DAD* Diffuse alveolar damage, *MV* Mechanical ventilation, *SAPS* Simplified Acute Physiology Score II, *PEEP* Positive end-expiratory pressure, *VE* Minute ventilation, *PaCO*_*2*_ Partial pressure of arterial carbon dioxide, *PaO*_*2*_, Partial pressure of arterial oxygen, *PaO*_*2*_ */FiO*_*2*_ Ratio of partial pressure of arterial oxygen to fraction of inspired oxygen

After adjustment, no ancillary variables remained significantly associated with DAD (Table 2). Using multivariate logistic regression analysis, the four markers independently associated with DAD were (1) duration of evolution of ARDS of more than 3 days, (2) severe hypoxemia, (3) increased dynamic driving pressure, and (4) diffuse opacities involving the four quadrants on chest radiographs (Table 2). The extent of opacities involving only three quadrants did not remain significantly associated with DAD.

**Table 2:** Predictors of diffuse alveolar damage in patients with clinical criteria for acute respiratory distress syndrome

	Bivariate test, OR (95% CI), *p* value	Multivariate logistic regression, OR (95% CI), *p* value
Characteristics of the patients		
Female sex, *n* (%)	1.79 (1.15–2.78), *p* < 0.01	Not significant
Pneumonia, *n* (%)	1.54 (0.99–2.39), *p* = 0.05	Not significant
Duration of evolution of ARDS		
> 72 h	3.92 (2.43–6.37), *p* < 0.001	**3.29 (1.95–5.55),** ***p*** **< 0.001**
Severity of hypoxemia	<0.0001	
Mild ARDS (*n* = 49)	Reference	
Moderate ARDS (*n* = 141)	4.72 (1.88–11.8), *p* < 0.001	**3.92 (1.48–10.3),** ***p*** **= 0.006**
Severe ARDS (*n* = 166)	10.1 (4.06–24.9), *p* < 0.001	**6.18 (2.34–16.3),** ***p*** **< 0.001**
Ancillary variables of severity		
PEEP ≥ 10 cmH_2_O	2.33 (1.51–3.59), *p* < 0.001	Not significant
Dynamic compliance < 40 ml/cmH_2_O	5.12 (1.73–15.1), *p* < 0.01	Not significant
VE corrected PaCO_2_ ≥ 10 L/minute	2.34 (1.40–3.93), *p* < 0.01	Not significant
PaCO_2_ > 45 mmHg	1.73 (1.14–2.65), *p* = 0.01	Not significant
Dynamic driving pressure (peak − PEEP), cmH_2_O	1.06 (1.04–1.09), *p* < 0.001	**1.06 (1.04–1.09),** ***p*** **= 0.007**
Extent of infiltrates on chest radiograph	<0.0001	
Two quadrants	Reference	
Three quadrants	2.45 (1.26–4.76), *p* < 0.01	1.63 (0.79–3.39), *p* = 0.19
Four quadrants	5.43 (2.93–10.1), *p* < 0.0001	**2.91 (1.47–5.75),** ***p*** **= 0.002**

*Abbreviations*: *ARDS* Acute respiratory distress syndrome, *DAD* Diffuse alveolar damage, *PEEP* Positive end-expiratory pressure, *VE* Minute ventilation; PaCO_2_, Partial pressure of arterial carbon dioxide

All variables nonredundant associated with DAD with a *p* value < 0.15 were included in the maximal model, including severity score, sex, pneumonia as a risk factor for ARDS, duration of ARDS evolution > 72 h, severity of hypoxemia (mild, moderate, or severe using mild as the reference), PEEP ≥ 10 cmH_2_O, dynamic compliance of the respiratory system < 40 ml/cmH_2_O, expired minute volume corrected by PaCO_2_ ≥ 10 L/minute, hypercapnia defined as PaCO_2_ > 45 mmHg, dynamic driving pressure defined as peak pressure minus PEEP, and the extent of radiological infiltrates (two, three, or four quadrants using two quadrants as the reference)

Bold data are variables that remained significanltly associated with DAD after multivariate logistic regression

**Fig. 1:**
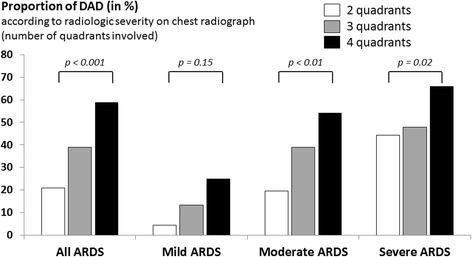
Bar graph showing the proportion of patients with diffuse alveolar damage (DAD) according to radiologic severity (extent of opacities on chest radiographs) in mild, moderate, and severe acute respiratory distress syndrome (ARDS). All in all, DAD was significantly more frequent in patients with extensive infiltrates involving the four quadrants than in the others: 101 (59%) of 171 patients with four quadrants vs. 58 (31%) of 185 patients with two or three quadrants (*p* < 0.001)

**Fig. 2:**
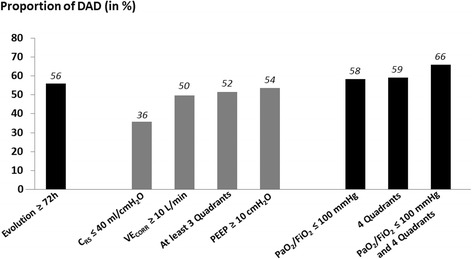
Bar graph showing the proportion of patients with diffuse alveolar damage (DAD) according to characteristics of the patients and criteria for severity of acute respiratory distress syndrome (ARDS). After multivariate analysis, the four variables independently associated with DAD (indicated by *black bars*) were duration of evolution of ARDS (≥3 days), severity of hypoxemia, increased dynamic driving pressure, and radiologic severity with diffuse opacities involving the four quadrants. *C*_*RS*_ Respiratory system compliance, *PaO*_*2*_ */FiO*_*2*_ Ratio of partial pressure of arterial oxygen to fraction of inspired oxygen, *PEEP* Positive end-expiratory pressure, *VE*_*CORR*_ Corrected expired volume per minute

DAD was significantly more frequent in patients with extensive infiltrates involving the four quadrants than in those involving two or three quadrants: 101 (59%) of 171 patients vs. 58 (31%) of 185 patients (*p* < 0.001) (Fig. 1). Among the patients with severe ARDS (PaO_2_/FiO_2_ ≤ 100 mmHg), DAD was found in 66% of patients with extensive opacities involving the four quadrants vs. 44% and 48% in those with opacities involving two or three quadrants, respectively (*p* = 0.02) (Fig. 2). Compared with patients without DAD, those with DAD were more likely to die as a result of refractory hypoxemia: 44 (28%) of the 159 patients vs. 27 (14%) of 197 patients (*p* = 0.03).

**Fig. 3:**
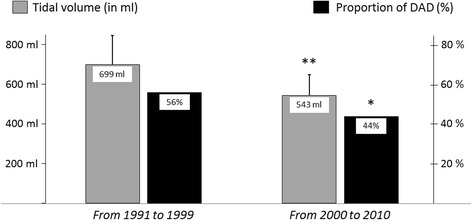
Bar graph showing temporal changes of tidal volume (*gray bars*) in patients with moderate or severe acute respiratory distress syndrome and the proportion of patients with diffuse alveolar damage (DAD) at autopsy examination (*black bars*) between the first decade at left (1991–1999) and the second decade at right (2000–2010) (i.e., before and after the era of reduction in tidal volumes). From 2000, tidal volumes were significantly reduced (from 699 ± 148 ml to 543 ± 106 ml, *p* < 0.0001), and this reduction was associated with a parallel reduction in the proportion of patients with DAD (from 56% to 44%, *p* = 0.03), suggesting that low tidal volumes may attenuate DAD. *p < 0.05, **p < 0.01

Patients with moderate or severe ARDS who died during the most recent period (between 2000 and 2010) received lower tidal volumes and were less likely to have DAD at autopsy examination than those from the earliest period (between 1991 and 1999) (Fig. 3).

## Discussion

### Key findings

In this large study of clinical autopsies performed in patients who fulfilled all the criteria for ARDS at the time of death, a typical histological pattern of DAD was observed in less than half of the patients and was significantly more frequent in patients with ARDS for > 3 days, in most patients with hypoxemia, in those with more increased dynamic driving pressure, and in patients with extensive opacities involving the four quadrants. Therefore, the degree of hypoxemia, the driving pressure, and the radiographic severity could be strong non-patient-dependent markers of DAD and could be easily assessed at the bedside.

### Markers of DAD

In addition to the degree of hypoxemia, the current Berlin definition proposes the following four ancillary variables to identify severe ARDS [16]: altered respiratory system compliance ≤ 40 ml/cmH_2_O, high minute ventilation corrected by PaCO_2_ ≥ 10 L/minute (indicating a large anatomical dead space), high PEEP levels ≥ 10 cmH_2_O, and radiologic severity with extensive opacities involving at least three quadrants. In a previous study, patients with DAD had a lower PaO_2_/FiO_2_ ratio and lower respiratory system compliance [20]. However, the extent of opacities on chest radiographs was not assessed therein. After multivariate analysis including radiographic severity, altered respiratory system compliance (<40 ml/cmH_2_O) did not remain significantly associated with DAD, whereas increased dynamic driving pressure and extensive opacities involving the four quadrants (and not three quadrants) constituted two factors independently associated with DAD in addition to hypoxemia severity.

We previously reported that DAD was markedly more frequent in most patients with hypoxemia, particularly after ≥ 3 days of evolution of ARDS [7]. However, in this previous study, we did not assess factors independently associated with DAD as we did in the present study. In addition to degree of hypoxemia and duration of evolution, we have now identified two other predictors of DAD: altered dynamic driving pressure and radiographic severity with diffuse opacities. Our data confirm that, even after adjustment for other variables, duration of evolution was independently associated with DAD. The absence of hyaline membranes during the first 3 days may be due to short duration of the process because hyaline membrane formation may take 2–3 days [2]. Moreover, patients with DAD had heavier lungs than the others, meaning that they had more alveolar and interstitial edema, one of the criteria for DAD. Although tidal volumes did not differ between patients with DAD and those without DAD, we found that the reduction of tidal volumes in the age of protective ventilation was associated with a parallel reduction of DAD in patients with moderate or severe ARDS, suggesting that low tidal volumes may attenuate DAD.

### Radiographic severity and clinical implications

It has been shown that patients with ARDS with diffuse opacities may differ from those with lobar opacities [21–24]. In a previous study, patients with diffuse ARDS were found to be more likely to have pulmonary ARDS, and also had lower respiratory compliance and higher mortality, than those with lobar ARDS [21]. The same authors found that lung morphology markedly influenced the effects of PEEP [22]. Indeed, PEEP induced marked alveolar recruitment in patients with diffuse opacities, whereas in patients with lobar opacities, PEEP induced mild alveolar recruitment associated with overdistention of previously aerated lung areas [22]. The authors suggested that in cases of lobar ARDS, there was mechanical loss of aeration in the lower lobes, whereas in cases of diffuse ARDS, an inflammatory mechanism may have occasioned aeration loss. In another study, effects of the prone position were more pronounced, with better oxygenation improvement in patients with lobar ARDS than in those with diffuse ARDS [23]. In a recent study, plasma biomarkers such as the soluble form of the receptor for advanced glycation end products or plasminogen activator inhibitor-1 were higher in diffuse ARDS than in lobar ARDS [24]. The high levels of these markers resulted from DAD and were associated with higher mortality. Consequently, patients with diffuse ARDS with extensive opacities involving the four quadrants could represent a homogeneous subphenotype of ARDS with behavior different from the others. Recently, Calfee and colleagues described several subphenotypes of ARDS that may have different responses to therapeutic strategies [25, 26]. In a subanalysis of a randomized controlled trial, the authors found that high PEEP levels decreased mortality only in a subgroup of patients characterized by particularly severe inflammation [26]. These patients were more likely than others to experience shock, metabolic acidosis, and higher levels of inflammatory biomarkers, and their outcomes were noticeably worse. ARDS is a syndrome involving highly heterogeneous patients, and identification of homogeneous subphenotypes could have an impact on the effects of different therapeutic strategies.

### Limitations

Obviously, clinical autopsy allows for analysis only of patients who have died, and therefore previously they have been among the most severely ill. Nearly half of the patients had severe ARDS at the time of death, and histological findings may differ from those in patients with less severe ARDS. However, in only 20% of cases was refractory hypoxemia the main reason for death. Although a high proportion of patients indeed died with ARDS, the proximate cause of death was septic shock, cardiac arrest, or withdrawal of life support. Because the latter was not always directly related to ARDS, these patients did not present the most pronounced respiratory severity at the time of death. In fact, they were in different stages of respiratory severity at the time of death, including 14% of those with mild ARDS, which means that our result could remain generalizable to patients with less severe ARDS. By contrast, open lung biopsy series included a subgroup of patients who may not have been representative; in fact, they were limited to those with persistent ARDS beyond 5–7 days without an identified cause [8–14], which represented only a small proportion of patients with ARDS.

Our study was conducted in a single center and over a lengthy period of time, at a time when ventilator strategies were markedly evolving, especially after 2000 and the widespread use of low tidal volumes [27–29]. A major limitation is that we could not collect accurate tidal volumes in milligrams per kilogram of predicted body weight over the whole period of the study, and consequently, although reduction in tidal volumes was associated with a parallel reduction in the incidence of DAD, we cannot assert that high tidal volumes really promote DAD. Similarly, we could not collect records of plateau pressure in all patients, and we calculated dynamic instead of static compliance and static driving pressure.

Another limitation was due to interobserver variability in the analysis of chest radiographs [30, 31], which may have led to misinterpretation of bilateral opacities. Normal lungs were found in 8% of the patients at autopsy examination, meaning that bilateral opacities seen on chest radiographs were probably atelectasis recruited during inflation at high pressure before microscopic analysis. In a previous study in which researchers compared the accuracy of chest radiography with computed tomography for the diagnosis of ARDS, many patients with ARDS based on computed tomography were detected using chest radiography [32]. However, sensitivity was higher for diffuse ARDS than for focal ARDS, and consequently diffuse opacities involving the four quadrants seem likely easier to identify.

DAD proportion may depend on the definition of ARDS that is being applied. In our study, the proportion of DAD was only 45% among all patients fulfilling the criteria for ARDS according to the Berlin definition (i.e., using “bilateral opacities” as radiologic criteria involving two, three, or four quadrants). In a previous study, the proportion of DAD reached 66% among all patients fulfilling the criteria for ARDS according to the American-European Consensus Conference definition and using diffuse opacities involving the four quadrants as radiologic criteria [4]. These two findings confirm the higher proportion of DAD among the most hypoxemic patients and those with diffuse infiltrates.

## Conclusions

In this large-scale study of clinical autopsies, a typical histological pattern of DAD was observed in only 45% of patients with ARDS. The four markers independently associated with the presence of DAD were duration of ARDS > 3 days, severe hypoxemia, more increased dynamic driving pressure, and extensive opacities involving the four quadrants. Radiographic severity was a strong marker of DAD easily assessed at the bedside. Whether these patients may require specific therapeutic strategies is a matter requiring further investigation.

## Abbreviations

ARDS: Acute respiratory distress syndrome
C_RS_: Respiratory system compliance
DAD: Diffuse alveolar damage
FiO_2_: Fraction of inspired oxygen
ICU: Intensive care unit
MV: Mechanical ventilation
PaCO_2_: Partial pressure of arterial carbon dioxide
PaO_2_: Partial pressure of arterial oxygen
PaO_2_/FiO_2_: Ratio of partial pressure of arterial oxygen to fraction of inspired oxygen
PEEP: Positive end-expiratory pressure
SAPS II: Simplified Acute Physiology Score II
VE: Minute ventilation
VE_CORR_: Corrected expired volume per minute
